# Alternative Methods of Determining Hamstrings-to-Quadriceps Ratios: a Comprehensive Review

**DOI:** 10.1186/s40798-019-0185-0

**Published:** 2019-03-25

**Authors:** Cassio V. Ruas, Ronei S. Pinto, G. Gregory Haff, Camila D. Lima, Matheus D. Pinto, Lee E. Brown

**Affiliations:** 1 Centre for Exercise and Sports Science Research (CESSR), School of Medical and Health Sciences, Edith Cowan University, 270 Joondalup Dr, Joondalup, WA Australia; 2 Exercise Research Laboratory, School of Physical Education, Physioteraphy and Dance, Universidade Federal do Rio Grande do Sul, Rua Felizardo 750, Porto Alegre, RS Brazil; 3 Center for Sport Performance and Human Performance Lab, Department of Kinesiology, California State University, 800 N State College Blvd, Fullerton, CA USA

**Keywords:** Alternative H:Q ratios, Muscle imbalance, Risk of injury

## Abstract

The hamstrings-to-quadriceps muscle strength ratio calculated by peak torque has been used as an important tool to detect muscle imbalance, monitor knee joint stability, describe muscle strength properties and functionality, and for lower extremity injury prevention and rehabilitation. However, this ratio does not consider other neuromuscular variables that can also influence the antagonist to agonist muscle relationship, such as torque produced at multiple angles of range of motion, explosive strength, muscle size, muscle fatigue, or muscle activation. The aim of this study was to comprehensively review alternative methods of determining the hamstrings-to-quadriceps ratio. These include ratios calculated by angle-specific torque, rate of torque development, muscle size, fatigue index, and muscle activation (measured by electromyography). Collectively, the literature demonstrates that utilizing alternative methods of determining the hamstrings-to-quadriceps ratio can be functionally relevant for a better understanding of the neuromuscular mechanisms underpinning the interaction of strength between hamstrings and quadriceps. However, there is insufficient evidence to recommend any of the alternative methods as sensitive clinical tools for predicting injury risk and monitoring knee joint integrity. Future longitudinal studies, along with injury incidence, are needed to further investigate all alternative methods of determining the hamstrings-to-quadriceps ratio. These have potential to offer insight into how athletes and the general population should be trained for performance enhancement and injury reduction, and may be used along with traditional methods for a thorough assessment of an individual’s H:Q muscle balance.

## Key Points

- Hamstrings-to-quadriceps ratios calculated by peak torque are important tools to detect knee strength imbalances and associated injury risk, but do not consider other neuromuscular variables that can also influence the antagonist to agonist muscle relationship.
- Alternative methods of determining hamstrings-to-quadriceps ratios based on angle-specific torque, rate of torque development, muscle size, fatigue index, and muscle activation have been proposed in the literature.
- Future longitudinal investigations are needed in order to recommend any of the alternative methods of determining hamstrings-to-quadriceps ratios as sensitive clinical tools for predicting injury risk and monitoring knee joint integrity. However, these have potential to offer insight into how athletes and the general population should be trained for performance enhancement and injury reduction.

## Background

The hamstrings-to-quadriceps (H:Q) muscle strength ratio has been used for more than 60 years to detect muscle imbalance, monitor knee joint stability, describe muscle strength properties and functionality, as well as serve as an important tool related to lower extremity injury prevention and rehabilitation [1–3]. Lower extremity injuries, such as anterior cruciate ligament (ACL) and hamstring strain, can occur when the hamstrings do not generate equivalent counter torque to decelerate rotation or high anterior tibial shear in extended knee movements, which are induced by quadriceps maximal torque [1–4]. This is the case of sports that present high rates of non-contact lower extremity injuries, such as soccer [2, 4, 5], American football [6], volleyball, and basketball [7], which makes H:Q ratio a common assessment performed by sport clubs for monitoring and training athletes [2, 4, 6, 8]. For instance, Croisier et al. [8], in a longitudinal study involving 687 professional soccer players, found that they were 4 to 5 times more likely to sustain hamstrings injuries when identified with low H:Q ratio and other strength imbalances during preseason. This is in agreement with Yeung et al. [9], who found that sprinters with H:Q strength imbalances during preseason had 17 times greater risk of sustaining hamstrings injuries during competition. Additionally, Li et al. [10] found that the restoration of H:Q strength balances by resistance training increased functionality of recreational athletes that had been arthroscopically diagnosed with a complete ACL tear. However, debate exists regarding the use of H:Q ratio as an injury predictive screening tool [11], as a few cohort studies have found weak or no association between H:Q ratio and lower-extremity injury risk [12–14]. The reasons for this may be related multifactorial nature of ACL tears and hamstring strains [14, 15], different use of predictive models and cut-off/normative values [2, 12, 16], as well as difficulty in validating robust screening tests to predict or prevent injuries [17].

The importance of this ratio was first introduced by Steindler [18], who suggested that a concentric peak torque (PT) H:Q torque ratio, known as conventional ratio (CR), that exceeded a magnitude of 3:2 (i.e., 0.66) was ideal during knee muscle force production. Heiser et al. [19] extended this rationale by reporting that the number of injuries of university soccer players started to decrease as soon as H:Q imbalances started to be monitored. This monitoring was used to ensure that soccer players reached CR of at least 0.6 by resistance training on an isokinetic dynamometer prior to return to play. However, the acceptance and use of this normative value by a variety of studies was first questioned by Nosse [20], who reported that the concept that hamstrings were 60% as strong as quadriceps was based on investigations measuring quadriceps and hamstrings isometrically by the use of a cable tensiometer in senior college football players. They reported that the H:Q relationship is actually in a wide range from 47–75% to 43–90% when tested on isotonic and isokinetic devices, respectively, in different populations. Therefore, it may be difficult to generalize normative values for the CR as it may vary across different population, range of motion (i.e., length/tension relationship), and velocity of the movement (i.e., force/velocity relationship) [20].

The CR is far from muscle functionality, as it does not consider the deceleration performed by eccentric torque during quadriceps concentric muscle actions [3, 4, 21–23]. This contention is based upon the work of Osternig et al. [24], who investigated the quadriceps electromyography (EMG) activation to hamstring coactivation mechanism in sprinters and distance runners. It was identified that hamstrings were much more eccentrically activated than quadriceps for limb deceleration purposes in both populations, indicating that the eccentric component of hamstrings (i.e., muscle tension) should be considered when assessing quadriceps isokinetic torques. However, Dvir et al. [25] were the first to describe the eccentric hamstring/concentric quadriceps PT ratio, also called dynamic control or functional ratio (FR), in patients that had complete tear of the ACL, being identified as having an increased H:Q ratio due to weakness or neural inhibition of quadriceps muscle activation. Aagaard et al. [3, 22] in two studies extended the use of this ratio for injury prevention in athletes, proposing that an FR of 1.0 characterized an appropriate balance. They suggested that this relationship ensured appropriate balance even at increasing isokinetic velocities and knee extended joint angles, which showed that hamstrings eccentric strength is essential for maintaining knee joint stability during knee extensors concentric strength. Therefore, the most common recommendation for reducing knee strength imbalance and injury risk is to increase hamstrings eccentric strength to equalize concentric quadriceps strength [2, 3, 8, 22, 26].

The H:Q ratio can be affected by main variables, such as dynamic and isometric muscle actions, and angle and velocity specificity [1, 3, 27–31]. For instance, CR and FR are based on dynamic antagonist and agonist PT values that are found in non-corresponding angles [1, 5, 27, 29, 32, 33]. Therefore, the H:Q ratio calculated at specific angles may be a more accurate estimation of potential knee strength imbalances [1, 5].

Additionally, both CR and FR are generally increased with increases in the isokinetic velocity used for testing concentric and eccentric muscle actions. This may occur because hamstrings concentric maximal strength (ratio numerator) is greater than quadriceps concentric maximal strength (ratio denominator) at high velocities, increasing the CR [34]. However, the FR increases at faster velocities because the hamstrings maximal eccentric strength, which is the ratio numerator, increases or remains the same while the quadriceps maximal concentric strength, which is the ratio denominator, decreases [31].

Furthermore, the H:Q ratio calculated by PT does not consider other neuromuscular variables that can influence the antagonist to agonist muscle relationship, such as torque produced at multiple angles of range of motion (ROM), explosive strength, muscle size, muscle fatigue, or muscle activation. Therefore, alternative methods of determining H:Q ratio that take into account these variables have been proposed in the literature. These include ratios calculated by angle-specific torque (AST), rate of torque development (RTD), muscle size (MS), fatigue index (FI), and muscle activation (MA) (measured by EMG). However, the importance, clinical, and practical relevance of these alternative H:Q ratio methods are not fully understood. Therefore, the aim of this literature review is to review alternative methods of determining the H:Q ratio as a measure of knee muscle strength balance. This will investigate the importance of alternative H:Q ratios as sensitive screening tools to predict injury risk and monitor joint integrity in athletes and general population.

## Literature Search Methodology

This review was based on 27 studies, published between 1998 and 2018 [3, 5, 27–30, 33, 35–54]. The articles were found by accessing the databases PubMed, Web of Science, and SPORTDiscuss using the following search terms: “Alternative” or “Angle Specific Torque” or “Rate of Torque/Force Development” or “Muscle Size/Muscle Thickness/Cross Sectional Area” or “Fatigue/Fatigue Index/Muscle Endurance” or “Muscle Activation/EMG” and “Hamstrings-to-Quadriceps Ratio” (e.g., “Muscle Size Hamstrings-to-Quadriceps Ratio”). To be selected, articles had to include the calculation of H:Q ratio involving at least one of these variables in the methods section. Only studies in the English language were considered. Articles that did not match these terms were excluded. Forty-four [1, 2, 4, 6–26, 31, 32, 34, 55–71] additional articles were used to support the variables investigated in the introduction and discussion of this review. Search included articles that had H:Q ratio calculations based on isokinetic dynamometry, magnetic resonance imaging (MRI), B-mode ultrasound, and EMG measurements. Due to the novelty in the use of some of the H:Q ratio methods, abstracts included in recent annals of journals were considered only if crucial for discussing some of the topics. Searches were ceased on September, 2018. Five topics were discussed based on the results of the search: angle-specific torque H:Q ratio; rate of torque development H:Q ratio; muscle size H:Q ratio; fatigue index H:Q ratio; and muscle activation H:Q ratio. The aims, sample, methodology, and results of each selected study are presented in Table 1. The measurements and calculations used by the selected studies for determining the alternative H:Q ratios are described on Table 2.

**Table 1:** Summary of studies

Topic	Authors	Aims related to H:Q ratio	Sample	Main outcome measures	Main results
AST H:Q ratio	Eustace et al. 2017 [37]	To compare PT and AST H:Q ratio (FR) between limbs, angles and velocities	26 male professional soccer players	PT H:Q ratio and AST H:Q ratio at 10° angles from 40 to 70° (0° = full knee extension) at three different isokinetic velocities (60, 180, and 270°/s) on the dominant and non-dominant legs	PT H:Q ratio was greater for the dominant than the non-dominant leg, and increased with higher velocities. AST H:Q ratio was greater for the dominant than the non-dominant leg at angles 60°, 50°, and 40°
	Eustace et al. 2018 [38]	To compare PT and AST H:Q ratio (FR) between senior professional and youth soccer players	17 senior professional and 17 elite youth male soccer players	PT H:Q ratio and AST H:Q ratio at ten degree angles from 40 to 70° (0° = full knee extension) at three different isokinetic velocities (60, 180 and 270°/s) on the dominant and non-dominant legs	PT H:Q ratio was greater for the dominant than the non-dominant limb and increased with higher velocities similarly for both groups. AST H:Q ratio was greater at 70° and 60° on the non-dominant leg for youth compared to senior players
	Aagaard et al. 1998 [3]	To assess PT and AST H:Q ratio (CR and FR) at different angles and velocities	9 track and field athletes (5 males and 4 females)	PT H:Q ratio and AST H:Q ratio at 50°, 40°, and 30° of knee flexion (0° full knee extension) at two different isokinetic velocities (30 and 240°/s)	PT and AST H:Q ratio (FR) increased across movement velocities and at extended knee joint angles. AST H:Q ratio (CR) increased across extended knee joint angles. PT H:Q ratio (CR) was not affected by velocity
	El-Ashker et al. 2017 [27]	To investigate sex differences on PT and AST H:Q ratio (FR)	96 young adults (50 males and 46 females)	PT H:Q ratio and AST H:Q ratio at 15°, 30°, and 45° of knee extension (0° = full knee extension) at three different isokinetic velocities (60, 180 and 300°/s)	PT H:Q ratio was lower for females compared to males. PT and AST H:Q ratio decreased for both groups with increased isokinetic velocity and close to full knee extension
	Cohen et al. 2015 [28]	To investigate the effect of simulated soccer on PT and AST H:Q ratio (FR)	9 semi-professional male soccer players	PT H:Q ratio and AST H:Q ratio at 9 knee flexion angles (from 10 to 90°; 0° = full knee extension) at 120°/s	Simulated soccer led to reduced PT H:Q ratio. Additionally, there was an AST H:Q ratio decrease at 10° of knee flexion
	Hiemstra et al. 2004 [36]	To compare PT and AST H:Q ratio (CR and FR) at different angles, velocities and contraction types between hamstring tendon ACL reconstruction and uninjured control participants	16 participants (9 males and 7 females) with more than 1 year of hamstring tendon ACL reconstruction and 30 active uninjured males	PT H:Q ratio and AST H:Q ratio measured from 5 to 95° of knee flexion (0° = full knee extension) at five angular velocities (50, 100, 150, 200, and 250°/s). *Strength maps* were used for comparisons between groups	Participants with ACL reconstruction had overall lower PT H:Q ratio compared to active participants, and presented lower AST H:Q ratio near full knee flexion and higher AST H:Q ratio near full knee extension
	Huang et al. 2017 [33]	To investigate PT and AST H:Q ratio (CR and FR) in participants with unilateral and symptomatic ACL deficiency	46 male participants with unilateral chronic ACL-rupture	PT H:Q ratio and AST H:Q ratio at 6 knee flexion angles (from 30 to 80° of knee flexion; 0° = full knee extension) on ACL deficiency and uninvolved (healthy) limbs at 60°/s	There was no difference between groups for PT H:Q ratio. ACL deficiency limbs presented greater AST H:Q ratio compared to healthy limbs at 30° and 40° of knee flexion
	De Ste Croix et al. 2017 [29]	To investigate sex differences on PT and AST H:Q ratio (FR) measured at different angles and velocities	55 male and 55 female recreationally active participants	PT H:Q ratio and AST H:Q ratio at three knee flexion angles (15, 30, and 45°, 0° = full knee extension) and three angular velocities (60, 120, and 240°/s)	PT H:Q ratio was lower at 120°/s and 240°/s and AST H:Q ratio was lower at 15° and 30° of knee flexion in females compared to males
	Ayala et al. 2012 [35]	To determine the absolute reliability of the PT and AST H:Q ratio (CR and FR)	50 recreational athletes (26 males and 24 females)	PT H:Q, AST H:Q ratio at three different joint angles (10°, 20°, 30°), and ROM-specific torque H:Q ratios at 4 different ranges (0–10°, 11–20°, 21–30°, and 0–30°) of knee flexion measured on three different occasions	PT H:Q ratio presented moderate reliability values. AST and joint ROM-specific torque H:Q ratios demonstrated poor absolute reliability scores
	Evangelidis et al. 2015 [5]	To compare PT, isometric, and AST H:Q ratio between soccer players and recreationally active males	10 soccer players and 14 recreationally active males	PT H:Q ratio, isometric H:Q ratio at five angles (105°, 120°, 135°, 150°, and 165°) and AST H:Q ratio at five angles (from 100 to 160° for 60°/s; from 105 to 160° for 240°/s; and from 115 to 145° for 400°/s)	PT, isometric and AST H:Q ratios were similar between professional soccer players and recreationally active players at any velocity
RTD H:Q ratio	Zebis et al. 2011 [45]	To compare the RTD H:Q ratio with isometric H:Q ratio	23 elite soccer players (11 females and 12 males)	Isometric H:Q ratio and RTD H:Q ratio at incrementing time periods of 10 ms (from 0 to 250 ms)	RTD H:Q ratio from 0 to 50 ms was lower than isometric H:Q ratio
	Greco et al. 2012 [39]	To compare PT H:Q ratio (CR) and RTD H:Q ratio in soccer players with high and low strength levels	39 male professional soccer players	PT H:Q ratio and RTD H:Q ratio calculated at a time interval of 0–50 ms	Soccer players who had high strength levels had greater PT H:Q ratio and RTD H:Q ratio compared to counterparts with low strength levels. There was no correlation between ratios
	Hannah et al. 2014 [41]	To compare isometric H:Q ratio and RTD H:Q ratio	20 untrained males	Isometric H:Q ratio and RTD H:Q ratio at time intervals of 25, 50, 75, 100, and 150 ms of force onset	RTD H:Q ratio was lower than isometric H:Q ratio
	Hannah et al. 2015 [30]	To investigate sex differences on isometric H:Q ratio and RTD H:Q ratio	40 untrained adults (20 males and 20 female)	Isometric H:Q ratio and RTD H:Q ratio calculated at time intervals of 25, 50, 75, 100, and 150 ms of force onset	Isometric H:Q ratio was greater in males than females. RTD H:Q ratio was similar between sexes at each time point
	Jordan et al. 2015 [42]	To investigate RTD H:Q ratio in elite ski racers	29 elite alpine ski racers (13 males and 8 females uninjured and 3 males and 5 females with ACL reconstruction)	RTD H:Q ratio calculated at time intervals of 0–50, 0–100, 0–150, and 0–200 ms	Elite alpine ski racers with ACL reconstruction had greater RTD H:Q ratio at 0-50 ms compared to uninjured counterparts
	Palmer et al. 2017 [43]	To examine the effects of age on isometric H:Q ratio and RTD H:Q ratio	15 young and 15 older women	Isometric H:Q ratio and RTD H:Q ratio at time intervals of 0–30 and 0–200 ms	Older women had greater RTD H:Q ratio at 0–200 ms compared to young women
	Greco et al. 2013 [40]	To investigate the effect of a fatiguing soccer-specific exercise on H:Q ratio (CR and FR) and RTD H:Q ratio	22 male professional soccer players	PT H:Q ratio at 180°/s and RTD H:Q ratio at time intervals of 0–50 and 0–100 ms	The fatiguing soccer-specific exercise led to lower PT H:Q ratio, but there was no effect on RTD H:Q ratio
	Thorlund et al. 2008 [44]	To investigate the effect of a simulated handball match on isometric H:Q ratio and RTD H:Q ratio	10 male elite handball players	Isometric, and RTD and RTD impulse H:Q ratios at time intervals of 30, 50, 100, and 200 of force onset	The simulated handball match did not lead to changes on isometric H:Q ratio, but RTD and RTD impulse H:Q ratios increased at 0–30 ms
FI H:Q ratio	Pinto et al. 2018 [52]	To investigate the influence of neuromuscular fatigue on PT H:Q ratio (CR) and the association between FI H:Q ratio and PT H:Q ratio	35 male elite professional soccer players	PT H:Q ratio and FI H:Q ratio at 300°/s	FI H:Q ratio was greater and weakly correlated to PT H:Q ratio. PT H:Q ratio only declined in the last three repetitions of the fatigue test, which was strongly correlated to hamstring torque decreases, but weakly correlated to quadriceps torque decreases
	Costa et al. 2018 [51]	To investigate the effect of hamstrings stretching and fatigue on H:Q ratio (CR) and FI H:Q ratio	35 healthy adults (17 females and 18 males)	PT H:Q ratio and FI H:Q ratio at 180°/s	There was no effect of combined stretching and fatigue on PT and FI H:Q ratios
MS H:Q ratio	Denadai et al. 2016 [47]	To investigate the association between PT H:Q ratio (CR) and MS H:Q ratio	9 male professional soccer players	PT H:Q ratio at 60°/s and MS H:Q ratio	PT H:Q ratio did not significantly correlate with MS H:Q ratio
	Evangelidis et al. 2016 [48]	To investigate the association between PT H:Q ratio (FR), isometric H:Q ratio and MS H:Q ratio	31 recreationally active young men	PT H:Q ratio at 50 and 350°/s, isometric PT H:Q ratio, and MS H:Q ratio	MS H:Q ratio was positively correlated with isometric H:Q ratio and PT H:Q ratio at both velocities
	Wieschhoff et al. 2017 [50]	To compare a MS VM:SM ratio between patients with ACL tears and participants with no ACL abnormalities	100 ACL tear and 100 control participants without ACL abnormalities (54 male and 46 female in each group)	MS VM:SM ratio	MS VM:SM ratio of patients that had recent non-contact ACL injuries was greater than control participants without ACL abnormalities
	Behan et al. 2018 [46]	To investigate sex differences on MS H:Q ratio	66 healthy active young (32 males and 34 females)	MS H:Q ratio	MS H:Q ratio was lower in females compared to males
	Ruas et al. 2017 [49]	To compare 3 different resistance training protocols involving concentric and eccentric muscle actions on MS H:Q ratio	Forty untrained males	MS H:Q ratio	No resistance training protocol led to any changes on MS H:Q ratio
MA H:Q ratio	Aagaard et al. 2000 [53]	To investigate the amount of antagonist coactivation during maximal quadriceps contraction and assess the MA H:Q ratio	60 sedentary males	MA (EMG-moment) H:Q ratio at 30°/s	Hamstrings coactivation and MA H:Q ratio were increased towards full knee extension
	Kellis and Katis 2007^a^ [54]	To investigate the AST H:Q ratio (FR) and the MA H:Q ratio at different velocities and movement directions	17 pubertal males	AST and MA (EMG-moment) H:Q ratio from 0 to 90° of knee flexion at 60°/s and 180°/s	AST H:Q ratio increased as the knee extended and at an increased angular velocity. MA H:Q ratio increased near full extension

*AST* angle-specific torque, *RTD* rate of torque development, *FI* fatigue index, *MS* muscle size, *MA* muscle activation, *EMG* electromyography, *PT* peak torque, *ACL* anterior cruciate ligament, *VM* vastus medialis, *SM* semimembranosus, *ROM* range of motion, *H*:*Q ratio* hamstrings to quadriceps ratio, *CR* conventional ratio, *FR* functional ratio

^a^Article was also discussed in topic: AST H:Q ratio

**Table 2:** Alternative H:Q ratios used by studies

Alternative H:Q ratio	Measurement	Calculation
AST H:Q ratio	Maximal concentric and eccentric hamstring and quadriceps strength are measured on an isokinetic dynamometer. Tested hamstring and quadriceps ROM are divided into several corresponding degree angles (e.g., 10, 20, 30...90° of knee flexion) or joint ranges (e.g., 0–10°, 10–20°, 20–30° of knee flexion). Hamstring and quadriceps concentric and eccentric AST are measured at each angle or by the PT of each joint range	CR: Hamstring concentric AST ÷ quadriceps concentric AST FR: Hamstring eccentric AST ÷ quadriceps concentric AST
RTD H:Q ratio	Maximal and/or explosive hamstring and quadriceps isometric strength is measured on an isokinetic/custom built dynamometer. RTD is measured by the slope of the torque-time curve of specific time intervals from the force onset (e.g., 0–50, 0–100, 0–150 ms)	Hamstrings RTD ÷ quadriceps RTD (at each time interval)
FI H:Q ratio	Several maximal concentric hamstring and quadriceps strength repetitions (e.g., 30 and 50) are performed on an isokinetic dynamometer at a high angular velocity (e.g., 150°/s and 300°/s). FI can be calculated by different methods involving the difference between individual, PT or mean of the final and initial repetitions [e.g. (initial PT - final PT) ÷ initial PT × 100]	Hamstring FI ÷ quadriceps FI
MS H:Q ratio	Hamstring and quadriceps MS are measured by ACSA or MT obtained from MRI or ultrasound scans. The muscle average area values, the sum of MT or the volume by cubic spline interpolation of hamstrings and quadriceps muscles are calculated to determine MS H:Q ratio	Hamstring MS ÷ quadriceps MS
MA H:Q ratio	Maximal concentric quadriceps strength is measured on an isokinetic dynamometer. Hamstring and quadriceps muscles are fitted with EMG electrodes to measure MA during the test, allowing the MA H:Q ratio calculation. The EMG-moment relationship can also be calculated in order to account for the influence of moment during co-activation and provide a more realistic analysis of EMG H:Q ratio. For this, antagonist muscle moments can be predicted by EMG-to-moment algorithm or second order polynomial	Hamstring EMG ÷ Quadriceps EMG or Hamstring EMG-moment ÷ quadriceps EMG-moment

*AST* angle specific torque, *RTD* rate of torque development, *FI* fatigue index, *MS* muscle size, *MT* muscle thickness, *ACSA* anatomical cross-sectional area, *MA* muscle activation, *EMG* electromyography, *PT* peak torque, *ROM* range of motion, *H*:*Q ratio* hamstrings to quadriceps ratio, *CR* conventional ratio, *FR* functional ratio

## Main Text

### Angle-Specific Torque H:Q Ratio

AST H:Q ratio has been used as an alternative method for determining knee strength imbalances, since PT values are usually found in non-corresponding antagonist-agonist angles and do not assess muscle balance through the entire test ROM [1, 5, 27, 29, 33]. This ratio has been implemented as a method for measuring the knee strength balance necessary for maintaining knee joint stability especially at end points of joint ROM due to vigorous eccentric contraction of the antagonist to decelerate fast speed agonist movements [5, 27, 29, 33]. Therefore, AST H:Q ratio may provide precise information on unilateral strength imbalances at corresponding angles through the entire ROM [1, 27]. Additionally, the AST can also be used to determine specific angles where side-to-side strength differences between limbs are present [63]. For the calculation of this ratio, the tested quadriceps and hamstrings ROM is divided into several corresponding degree angles (e.g., 10, 20, 30...90° of total ROM) or joint ranges (0–10°, 10–20°, 20–30° of total ROM) [1, 5, 27, 29, 33, 35].

Aagaard et al. [3] calculated AST H:Q ratio (FR) at 30°, 40°, and 50° through 80° of knee ROM (0° = full extension) in track and field athletes. They found that this ratio increased at more extended angles to a proportion greater than 1.0 from slow to fast speeds. This may be because when the hamstring is at optimal length for force production, the quadriceps length is at a reduced force generating capacity. In agreement to this, Kellis and Katis [54] found that AST H:Q ratio (FR) calculated at every 10° knee angle increased significantly toward knee extension at slow and fast velocities, and exceeded 1.0 especially at the fastest velocity in seventeen pubertal males. The authors also concluded there was an influence of velocity on the H:Q ratio because as velocity increases the concentric moment of the quadriceps declined, while the hamstrings eccentric moment was unaltered. Eustace et al. [37, 38] in two studies also identified the importance of the use of AST rather than PT single angle metrics to measure H:Q ratio for injury prevention. They found that AST H:Q ratio can vary across velocities, angles, limbs, and professional experience in soccer players and give greater insight into lower extremity muscle and ligament injuries that typically occur in extended knee angles and at high velocities. Contrary to these findings, El-Ashker et al. [27] found that hamstrings had lower eccentric torque production compared to quadriceps concentric torque near full knee extension in healthy adults, leading to decreased AST H:Q ratio (FR). They suggested that the low AST H:Q ratios near full knee extension may represent the angles where injury is most likely to occur. The authors concluded that differences between their findings compared to previous studies may have resulted because of the reduced number of angles analyzed in the determination of the AST H:Q ratio, as well as participants potentially not fully resisting the dynamometer’s lever arm during the eccentric maximal strength test. However, Cohen et al. [28] found that AST H:Q ratio (FR) at extended angles decreased and PT shifted to a shorter angle after intermittent fatiguing activities in soccer players. They concluded that prevention and rehabilitation resistance training programs should focus on strengthening and increasing fatigue resistance on angles close to full knee extension.

However, only a few studies have attempted to demonstrate a systematic association between reduced AST H:Q ratio at extended knee angles and increased muscle and ligament injury rate. For instance, Hiemstra et al. [36] found that male participants recovering from hamstring tendon ACL had lower AST H:Q ratio (CR and FR) compared to active participants tested through 90° of knee ROM. Results also demonstrated that participants with ACL reconstruction have increased alteration of muscle balance during knee flexion-extension motion, as they have reduced ability to stabilize the knee joint at extreme knee flexion, but attempt to heavily rely on hamstrings during full knee extension [36]. In agreement to this, Huang et al. [33] found that ACL ruptured limbs presented greater AST H:Q ratio (FR and CR) compared to healthy limbs only at 30° and 40° of knee flexion (0° = full knee extension). This demonstrates that ACL injuries may lead to a quadriceps strength and activation deficit at more extended knee angles, increasing the H:Q ratio. Therefore, monitoring muscle balance through AST H:Q ratio has been recommended after ACL reconstruction to create postoperative rehabilitation programs to reduce imbalances in primary angles that could lead to re-injury [36].

Nevertheless, it has been proposed that AST H:Q ratio should be used with caution as it may vary among sex [29] and is less reliable than traditional H:Q ratio measurements [35]. De Ste. Croix et al. [29] found that AST H:Q ratio (FR) was significantly lower for women compared to men at the two angles closer to full knee extension (i.e., 15° and 30°; 0° = full extension), demonstrating that women may have increased injury risk compared to men due to their inability to recruit the entire motor unit pool during eccentric maximal strength production at these angles, affecting dynamic knee stability. Ayala et al. [35] questioned if AST H:Q ratio was in fact sensitive enough to be used for monitoring clinical goals during rehabilitation training programs as AST H:Q ratio (CR and FR) had poor intra session reliability [percentage change in the mean (CM): − 14–12%; typical percentage error (CV_TE_): 19–61%; intraclass correlations (ICC): 0.1–0.6], compared to PT H:Q ratio (CM < 2.5%; CV_TE_: 16–20%; ICC: 0.3–0.7] when tested at different isokinetic velocities in active young adults on three different testing sessions, separated by 72–96-h interval. Although it was not possible to generalize these results to clinical or sport populations, the authors recommended that the use of AST H:Q ratio requires greater familiarization of the concentric and eccentric isokinetic test to ensure participants apply/resist force throughout full ROM, to reduce torque variability at extreme angles. Evangelidis et al. [5] also found that using AST H:Q ratio as a single measure of muscle injury risk may lead to erroneous conclusions, as AST H:Q ratio (CR and FR) measured at 5° intervals was similar between professional soccer players (who usually have high risk of hamstring strain injuries) and recreationally active players. They concluded that injury risk factors are multifactorial and other neuromuscular variables involved in regular exposure to soccer matches and training can increase injury rate during the practice of soccer.

Therefore, AST H:Q ratio can provide specific information regarding imbalances in knee strength through the entire ROM compared to PT H:Q ratio. However, the use of this ratio is relatively new, and there is not enough evidence to support its use as a lower-extremity injury risk prediction tool. Additionally, since AST H:Q ratio usually differs in relation to ROM joint angle or joint range, there is a need for specific normative values per angle to be created as a criterion to help to avoid and rehabilitate injuries. In order to increase H:Q ratio at specific angles, it may also be necessary to perform isometric resistance training to focus on the primary angles where injury is likely to occur. Therefore, longitudinal studies along with injury incidence, as well as preventive and rehabilitation resistance training investigations are necessary to further understand the most adequate use of AST H:Q ratio information in athletic and non-athletic populations.

### Rate of Torque Development H:Q Ratio

Most sports require the ability of the antagonist muscles to rapidly provide equivalent counter torque to decelerate agonist explosive movements. Additionally, ACL injuries are uncommon during heavy controlled lifts because the hamstrings have enough time to develop counter torque to decelerate the quadriceps and protect the knee joint [45]. Therefore, the determination of the rapid or explosive H:Q ratio has been introduced to more closely approximate to sport movements [39, 45]. This ratio is calculated by determining the hamstring RTD/quadriceps RTD, which is measured as the slope of the torque-time curve of specific intervals (e.g., 0–50, 0–100, 0–150 ms) [39, 45]. To our knowledge, Zebis et al. [45] was the first to introduce the use of this ratio in male and female soccer players, demonstrating that RTD H:Q ratio from 0 to 50 ms, calculated at every 10 ms, was lower than H:Q ratio calculated by isometric PT (i.e., isometric H:Q ratio, which usually takes > 300 ms), with no difference between sex. In addition, they recommended this ratio for clinical use as they found high reliability for RTD H:Q ratio. It was also reported that two female players with 37–48% lower RTD H:Q ratio compared to the sample mean experienced ACL injuries in the following season. Similarly, Greco et al. [39], demonstrated that soccer players who had high strength levels had 23% and 20% greater PT H:Q ratio (CR) and RTD H:Q ratio (0–50 ms) compared to counterparts with low strength levels. However, no correlation was found between both ratios, which showed that although PT and RTD have in common mechanisms in the early phase of contraction, H:Q ratio (CR) and RTD H:Q ratio should be analyzed separately as they may have dissimilar physiological and clinical meanings.

The rationale that RTD H:Q ratio assessment is needed for monitoring dynamic knee joint stability during fast sporting movements has also been supported by Hannah et al. [41], who found that RTD H:Q ratio at 0–50 ms was 56% lower than isometric H:Q ratio in untrained males. This may be because the hamstrings demonstrate increased electromechanical delay (EMD) when compared to quadriceps, resulting in a 21-ms delay in the onset of torque production. This delay may lead to instability and anterior tibial translation in the early phase of the contraction, increasing the risk of ACL injuries. In a follow up study, the same researchers found that although isometric H:Q ratio was greater for males than females, the RTD H:Q ratio was similar between sex [30]. This suggests that this ratio was not predictive of the usual higher injury rates reported for females compared to males in the practice of sports. Interestingly, after ACL reconstruction, this relationship seems to be inverted. For example, Jordan et al. [42] found that elite alpine ski racers with ACL reconstruction present greater RTD H:Q ratio at 0–50 ms compared to uninjured counterparts. Although no difference was found for RTD H:Q ratio interval times between 50 and 200 ms, the authors suggested caution to interpret these results in athletes with ACL reconstruction, as ligament injury may lead to quadriceps strength loss, inflating the RTD H:Q ratio. Similar results have been found in elderly women, who have been found to have a greater RTD H:Q ratio at 0–200 ms compared to young women (0.85 ± 0.25 vs. 0.62 ± 0.22) as a result of a greater age-related reduction in quadriceps than hamstrings RTD [43]. Since elderly women also demonstrated lower vertical jump power, a very high RTD H:Q ratio may actually reduce functional performance.

RTD H:Q ratio calculations can be a valid tool for calculating muscle imbalances in the early phases of sporting movements, such as in explosive ground contact situations, where several lower extremity non-contact injuries can occur [45]. However, RTD H:Q ratio is calculated based on quadriceps and hamstrings isometric explosive tests, which is far from the concentric and eccentric multi-joint dynamic performance seen in sports. For instance, Greco et al. [40] found that PT H:Q ratio (CR and FR) was reduced after a fatiguing soccer-specific exercise, which had no effect on RTD H:Q ratio at 0–50 ms and 0–100 ms. The authors concluded that fatigue may affect differently muscle mechanical properties between isokinetic and isometric muscle actions. Additionally, they reported that fatigue may reduce joint stabilization only at time intervals equivalent to reaching maximal torque. However, Thorlund et al. [44] found that H:Q ratio calculated by RTD and RTD impulse (i.e., area under the moment-time curve) were lower only at the very early phase of force (0–30 ms) after a simulated handball match. Although the authors did not discuss the results of the ratio further, they concluded that the overall decreases in jump height, MVC, RTD, and impulse at most time intervals from 0 to 200 ms, and EMG activity for the quadriceps and hamstring muscles during fatigue may potentially impair handball functional performance.

There are a few aspects that need to be considered when interpreting RTD H:Q ratio results. Since the calculation of this ratio does not consider hamstrings eccentric to quadriceps concentric strength, it does not totally reflect the knee strength balance required by sport activities. Additionally, both very low or very high RTD H:Q ratio may demonstrate a type of strength imbalance that could lead to decrements in functional activities. This demonstrates that RTD H:Q ratio needs to be analyzed along with raw RTD values for the best interpretation of potential knee imbalances and injury risk in the very early phase of movements.

### Fatigue Index H:Q Ratio

Several studies have found that the H:Q ratio decreases after fatiguing exercise [67–71], which may indicate that the hamstrings may fatigue earlier than the quadriceps during sporting activities due to their role in controlling and stabilizing the knee joint during lower limb movements. As a result, the increased H:Q ratio imbalance may potentially affect knee stability and increase the likelihood of muscle strains and ligament injuries [70]. In fact, a higher susceptibility of lower extremity non-contact injuries in elite soccer players has been reported to occur as a fatigue response during the last 15 min of the first and second halves of soccer matches [66]. This may be related to the lower capacity of hamstrings to perform eccentric deceleration of high velocity knee extension movements during the last third of matches [69, 71]. Additionally, Small et al. [71] found that H:Q ratio (FR) was reduced by ~ 15% after a 90-min soccer-specific aerobic field fatiguing protocol. Fatigue also led to a shift of optimum quadriceps and hamstrings concentric peak force production in the direction of longer muscle lengths. This may cause greater loss of concentric force at shorter muscle lengths, increasing susceptibility to muscle damage and injury risk. However, there was a shift of optimal hamstrings eccentric peak force production in the direction of shorter muscle lengths with fatigue. They concluded that this may explain the increased occurrence of hamstrings muscle strain injuries at more lengthened angles during the final stages of matches, when eccentric force production occurs at shorter muscle lengths.

A few studies have also used different fatiguing protocols on the isokinetic dynamometer to determine the exact moment where quadriceps and hamstrings force capabilities decline, resulting in reduced H:Q ratio. The FI is usually calculated by taking the average or highest PT of the initial repetitions, and the average or last PT of the final repetitions of a fatigue test [52, 62]. Sangnier et al. [64] tested university soccer players in a fatigue test involving 50 repetitions of dominant leg maximal concentric strength. They found that players presented a greater decline in hamstrings than quadriceps after 15 repetitions for the dominant limb, and after 40 repetitions for the nondominant limb. Additionally, the H:Q ratio (CR) from repetitions 30–50 for the dominant limb were 16–28% lower than the PT H:Q ratio (calculated by the mean of three maximal repetitions). A potential explanation for this may be that the hamstrings have a greater proportion of type II twitch fibers when compared to the quadriceps [64], are used less in activities of daily living, and are more prone to muscle damage and the effects of fatigue [52]. Similarly, Kawabata et al. [59] compared the PT H:Q ratio (CR) after three fatigue tests of 50 repetitions separated by 10 min rest between soccer players, baseball players, and marathon runners. They found that while hamstrings FI increased from 36–41%, 35–40%, and 13–18% by sport, respectively, quadriceps FI changed only slightly from 56–55%, 48–46%, and 40–43% throughout the series of tests for the same athletes, respectively. In addition, the PT H:Q ratio (CR) decreased more for soccer and baseball players, compared to marathon runners. Since marathon runners are endurance athletes, they are more resistant to fatigue as such displayed a different rate of change in the PT H:Q ratio. This suggests that fatiguing exercise (i.e., loss in force generating capacity) may affect the H:Q strength relationship differently, as H:Q ratio is likely to be differentially influenced by the type and intensity of exercise and the physical capabilities/phenotypes of athletes [2, 64].

To our knowledge, only two recent studies have attempted to use FI to calculate the H:Q ratio (i.e., hamstrings FI/quadriceps FI). Pinto et al. [52] found that the FI H:Q ratio (using several different calculations) ranged from 1.25 to 1.38, and significantly greater and weakly correlated to the PT H:Q ratio (CR) in professional soccer players. The PT of quadriceps and hamstrings gradually decreased from repetition 6 to repetition 30 (end of the test), but the PT H:Q ratio only declined in the last three repetitions. The H:Q ratio decline on repetitions 28–30 were strongly correlated to the decreases in hamstrings torque (*r* = 0.80–0.84). However, the H:Q ratio decline on repetition 29 was weakly correlated to decrease in quadriceps torque (*r* = 0.37). No correlations were found between repetitions 28 and 30 and quadriceps torque. The authors concluded that since soccer players have to perform sport-specific strength tasks (i.e., jumping, sprinting, kicking, etc.) under fatigue conditions, the FI H:Q ratio may be a better marker for injury risk and decisions regarding the return to play after injury. Costa et al. [51] examined the FI H:Q ratio measured across 50 repetitions of consecutive maximal unilateral isokinetic leg extension and flexion repetitions to assess the combined effects of hamstrings stretching and fatigue on the H:Q ratio in recreationally-active men and women. They found that the FI H:Q ratio from the combined stretching and fatigue condition was not significantly different than the non-stretching condition for men (~ 4%) and women (~ 52%). However, when the fatigue test was divided into five intervals of ten repetitions, the quadriceps PT decreased until the end of the test, while the hamstrings PT only declined until the 40th repetition in the stretching condition. Therefore, the authors concluded that stretching the hamstrings immediately prior to performing fatiguing activities may eventually cause earlier reductions in the torque capacity of this muscle group.

It seems clear that H:Q ratio can be affected by fatiguing activities, which are common due to the increased physical demand of most sports. Fatiguing exercises result in quadriceps and especially hamstrings loss of torque-generating capacity. Collectively, previous studies highlight that the potential reasons for this may be because hamstrings have a greater proportion of fast twitch fibers [64], greater shift of optimal eccentric peak force production in the direction of shorter muscle lengths during fatiguing exercise [71], and are less utilized in activities of daily living [52] compared to quadriceps. Since hamstrings control and stabilize the knee joint during lower limb actions, an H:Q ratio strength imbalance caused by fatigue may increase the possibility of knee ligament and muscle strain injuries. Based on this evidence, it has been recommended that resistance training programs focus on reducing the fatigue induced decreases in the H:Q ratio. One strategy to accomplish this goal is to increase hamstrings eccentric strength and resistance to fatigue with targeted resistance training interventions [58, 64]. Therefore, the FI H:Q ratio seems to be a promising muscle balance and injury prevention screening tool, as it accounts for muscle endurance capacity along with strength balance. Nevertheless, to date, no study has included hamstrings eccentric muscle actions on FI H:Q ratio calculations. Hamstrings eccentric muscle actions approximate functionality during athletic performance and may be more affected than hamstrings concentric torque during sporting activities [3, 4, 21–23]. Although a fatigue test that includes eccentric muscle actions performed by the hamstrings can expose participants to greater muscle damage, it may be worth using the FI H:Q ratio including muscle actions which compare the eccentric strength of the hamstrings to the concentric strength of the quadriceps. However, a method for testing FI H:Q ratio using eccentric muscle actions still needs to be validated in the scientific literature. Additionally, future research needs to examine if the FI H:Q ratio is predictive of injury thus monitoring studies are necessary to validate this hypothesis.

### Muscle Size H:Q Ratio

Increased MS H:Q ratio has been associated with the H:Q strength ratio, indicating that a reduced hamstrings relative to quadriceps MS may also lead to a H:Q muscle strength imbalance [48]. This has been suggested based on the potential positive influence of MS on maximal strength capacity [56, 61, 65]. Since maximal strength has been related to be influenced by morphological changes [47, 48], it is possible that the measurement of the balance between hamstrings and quadriceps MS can be an alternative method for measuring H:Q muscle balance and risk of injury [48].

Interestingly, the studies that have first suggested MS H:Q ratio measurement have found conflicting results when relating it to PT H:Q strength ratio. Denadai et al. [47] investigated MS H:Q ratio calculated by hamstrings and quadriceps anatomical cross-sectional area (ACSA) in professional soccer players with strength imbalance. This was characterized by PT H:Q ratio (CR) below a normative value of 0.6 in the dominant and non-dominant legs (e.g., ranging from 0.45–0.59). However, their results showed that for both legs, PT H:Q ratio did not significantly correlate with MS H:Q ratio. The authors concluded that PT H:Q ratio is not determined by MS H:Q ratio in this population, and that other neuromuscular variables such as neural drive, fiber-type composition, and tendon/aponeurosis stiffness could explain the strength imbalances of the soccer players of the study. Contrary to these findings, Evangelidis et al. [48] calculated quadriceps and hamstrings muscle volume by cubic spline interpolation and reported that MS H:Q ratio was positively correlated with the PT H:Q ratio (FR) measured at slow (50°/s, *r* = 0.34) and fast (350°/s, *r* = 0.56) isokinetic velocities in physically active men. Additionally, MS H:Q ratio explained 12–31% of the PT H:Q ratio. This was evidenced by quadriceps size explaining 30–31% of maximal concentric strength and hamstrings size explaining 48–58% of maximal eccentric strength. The PT H:Q ratio was also only influenced by knee flexors and not knee extensors. The authors concluded that H:Q MS H:Q ratio contributes to functional strength imbalances and that resistance training focusing on increasing hamstrings size and eccentric strength may be an appropriate strategy for injury prevention. The conflicting findings between Denadai et al. [47] and Evangelidis et al. [48] may be partially explained by the different sample sizes and populations, isokinetic velocities, and methods for evaluating MS H:Q in their studies.

Although a low MS H:Q ratio has been suggested to be related to injury incidence, to our knowledge, only one study has directly investigated its relationship to individuals with knee ligament injury. Wieschhoff et al. [50] investigated quadriceps to hamstrings CSA differences between participants with complete ACL tears and with no ACL abnormalities. In this study rather than ACSA of the entire muscle groups being investigated, vastus medialis (VM) represented the quadriceps and semimembranosus (SM) represented the hamstrings for the calculation of a VM:SM MS ratio. They found that VM:SM MS ratio of patients that had recent non-contact ACL injuries (≤ 7 days) was approximately 23% greater than the control group. The authors concluded that a high VM:SM MS ratio implies a deficiency in hamstrings strength and may be a risk factor for non-contact injuries resulting in ACL tears. Differently than typical H:Q MS ratio calculation, in this study, the ratio numerator was the quadriceps (VM), whereas the denominator was the hamstrings (SM), which may explain these results. In this investigation, there was no comparison of H:Q MS ratio between pre and post injury, which could lead to more comprehensive conclusions about its association with non-contact ACL tears. However, other confounding variables were controlled for, such as exclusion of patients with chronic ACL tears, time from injury to imaging, and recording of the mechanism of ACL injuries (contact vs. non-contact).

The authors also highlighted the need of future research demonstrating the effectiveness of targeted resistance training in reducing ACL injuries of participants presenting hamstrings reduced MS by imaging. Similarly, given the occurrence of greater incidence of ACL injuries in females than men, Behan et al. [46] investigated H:Q MS ratio in men and women. The authors found that females had 25.3% and 29.6% smaller quadriceps and hamstrings maximal ACSA resulting in a reduced H:Q MS ratio compared to men. They related their results to previous research demonstrating lower H:Q strength ratio in women than men, and highlighted the importance of hamstrings resistance training for injury prevention in females.

Most of the aforementioned studies indicate the need of longitudinal studies testing the effectiveness of resistance training for increasing the MS H:Q ratio and reducing injury risk. However, to our knowledge, only one study has attempted to compare MS H:Q ratio before and after resistance training. Ruas et al. [49] compared the effects of 6 weeks of training protocols with different muscle actions on MS H:Q ratio measured by muscle thickness via ultrasound in untrained males. Training groups performed either concentric quadriceps and concentric hamstrings, concentric quadriceps and eccentric hamstrings, eccentric quadriceps and eccentric hamstrings, or no training. The authors found that all training protocols elicited similar muscle thickness increases for hamstrings and quadriceps muscles, which did not lead to changes in H:Q MS ratio. They suggested that a greater volume of resistance training may be needed for hamstrings to elicit greater MS H:Q ratio.

Therefore, the literature demonstrates that since MS may partially explain hamstrings and quadriceps maximal strength production, the evaluation of MS H:Q ratio by imaging examination may identify decreased H:Q strength ratio and increased joint instability. However, it seems clear that future prospective studies are still needed to investigate the effect of targeted hamstrings and quadriceps resistance training on increasing MS H:Q ratio and reducing injury incidence. Additionally, although imaging techniques can be difficult and costly, the use of this ratio can benefit laboratories and clinics that focus research and practice on diagnostic imaging modalities. However, since no force measurement is considered in the calculation of this ratio, we believe caution should be taken when interpreting its results as a single measurement of H:Q muscle balance. Therefore, the use of H:Q MS ratio along with traditional H:Q ratio measured by PT may provide a more meaningful interpretation of H:Q muscle balance.

### Muscle Activation H:Q Ratio

Hamstrings muscle coactivation happens simultaneously to quadriceps muscle activation during knee extension motion to counteract the anterior shear of the tibia relative to the femur, thereby increasing joint stability and protecting the ACL [23, 33, 53, 54, 60]. However, all forms of H:Q ratio calculations utilize isolated moments of hamstrings and quadriceps, but not precisely the interaction of these two muscle groups during knee extension motion. Therefore H:Q ratio calculated by MA (measured by EMG) of hamstrings and quadriceps (also called H:Q coactivation ratio) have been proposed as an approach to simultaneously measure hamstrings coactivation to quadriceps activation during knee extension maximal strength [53, 54]. This information can give insight about risk of knee muscle and ligament injury, as well as improve ACL injury evaluation. Additionally, it may enable understanding mechanical and neurosensory functions of the ACL [53, 54].

To our knowledge, although previous studies have investigated the role of hamstrings coactivation on knee stability [23, 60], Aagaard et al. [53] was the first to introduce the MA H:Q ratio to quantify the proportion of hamstrings coactivation and moment of force during maximal quadriceps knee extension at 30°/s. The authors found that overall hamstrings coactivation was 15–35% relative to quadriceps activation in sedentary males, and that there was increased hamstrings coactivation toward full knee extension compared to the midpoint angles. They concluded that the increased coactivation of the hamstrings toward maximal concentric knee extension may be related to a mechanism to increase stability of the knee, preventing anterior tibial displacement and assisting in proprioceptive and mechanical roles of the ACL. Similarly, Kellis and Katis [54] found that MA H:Q ratio increased from 0.18 to 0.30 from knee flexion to near full knee extension. The authors reported that given the principle of reciprocal inhibition, the hamstrings may only generate the absolute necessary strength to decelerate and stabilize the knee. They concluded that a balanced coactivation between the two antagonistic muscle groups (i.e., hamstrings and quadriceps) is necessary for movement efficiency and joint stability during active knee extension. Therefore, both moment and EMG data (EMG-moment relationship) may provide useful information regarding knee muscle stability.

To our knowledge, no previous research has directly investigated if MA H:Q ratio can be used as a measurement to detect potential knee injury risk. Furthermore, neural inhibition during eccentric contractions may be a mechanism to protect against the high levels of forces produced by this muscle action [48, 55]. It may be that resistance training can alter the activation/inhibition processes between antagonistic muscles [47, 48], which may affect the H/Q coactivation ratio. Nevertheless, alterations in antagonist muscle activity have been highlighted as one of the most misunderstood adaptations to resistance training, as the central nervous system needs to optimize between force production (i.e., decrease in antagonist coactivation) and joint integrity (increase in antagonist coactivation) [57]. Therefore, given the importance of the coactivation mechanism between antagonistic muscles to maintain the integrity of the joint [57], the H:Q coactivation ratio may be a valuable tool to monitor neuromuscular activation for dynamic joint stabilization for injury prevention and rehabilitation purposes [54]. Further studies are needed to identify the validity of MA H:Q ratio in preventing for knee injury risk, as well as its changes due to resistance training.

## Conclusions

This review explored studies that have used alternative methods of determining H:Q ratio. Collectively, the studies demonstrate that utilizing alternative methods of determining H:Q ratio can be relevant for better understanding the neuromuscular mechanisms underpinning the interaction of strength between hamstrings and quadriceps. However, there is still not sufficient evidence to recommend any of the alternative methods of determining H:Q ratio as sensitive clinical tools for predicting injury risk and monitoring joint integrity. Additionally, our search indicated that the reliability of only two alternative methods of calculating H:Q ratio (AST and RTD) has been investigated. While AST H:Q ratio (CR and FR) was found to have poor inter session reliability [35], high reliability was found for RTD H:Q ratio [45]. Most studies that have proposed the use of alternative ratios have recommended resistance training interventions for increasing H:Q muscle balance. However, future longitudinal studies, along with injury incidence, are needed in order to validate specific normative values for each alternative H:Q ratio. These values could potentially be used as a target for injury prevention and rehabilitation in athletes and physically active individuals. If these concepts are further investigated, all alternative methods of determining H:Q ratio have potential to offer insight into how athletes and the general population should be trained for performance enhancement and injury reduction. These may be used along with traditional methods for a thorough assessment of an individual’s H:Q muscle balance.

## Abbreviations

ACL: Anterior cruciate ligament
ACSA: Anatomical cross sectional area
AST: Angle-specific torque
CR: Conventional ratio
EMD: Electromechanical delay
EMG: Electromyography
FI: Fatigue index
FR: Functional ratio
H:Q ratio: Hamstrings-to-quadriceps ratio
MA: Muscle activation
MRI: Magnetic resonance imaging
MS: Muscle size
PT: Peak torque
ROM: Range of motion
RTD: Rate of torque development
SM: Semimembranosus
VM: Vastus medialis
